# Before the Lords' Committee

**Published:** 1890-07-05

**Authors:** 


### Before the Lords' Committee.

VI.—SIR SYDNEY WATERLOW (*continued*).

The general effect of Sir Sydney Waterlow's evidence will be to discourage those medical reformers who desire to diminish the work of hospitals in order to increase that of family doctors. The witness brought out with striking clearness the fact that hospitals are powerful organisations; strong in material wealth, in established professional reputation, and in the determined and devoted loyalty both of their lay and professional representatives. If the final report of the Commission should run parallel or approximately parallel with the general lines of Sir Sydney's evidence, it is plain that the hardly-pressed practitioner must look elsewhere for help and deliverance than to the abatement of hospital competition. Sir Sydney, it is to be noted, is a strong witness, though he is not always scientifically impartial. He has had a long and intimate experience of the ways of hospitals, and he also knows a good deal about poor-law infirmaries, provident dispensaries, and other methods of charitable and semi-charitable medical relief. He has been chairman of the Distribution Committee of the Hospital Sunday Fund for many years; and this has given him opportunities of acquiring a broad and general comparative knowledge of all classes of hospitals within the metropolitan area.

On the burning question of the working of the out-patient department, Sir Sydney's evidence points to the conclusion that he is an ardent admirer of the status quo. According to him the out-patients at St. Bartholomew's Hospital last year numbered no more than 19,001. This extraordinary statement seemed to surprise the Commissioners a good deal, and they did not appear to quite appreciate the witness's distinction between out-patients and casualties, which latter class numbered as many as 137,399 last year. The Chairman and other noble lords returned again and again to this question, and seemed to find great difficulty in understanding what a casualty was if he were not an out-patient. As a matter of fact the word "out-patient" has come to have a generally accepted meaning, and it is ordinarily understood to include all those persons who receive treatment other than as in-patients, whether they receive that treatment once or oftener; whether they are casual patients or repeated callers. For practical purposes all who are not in-patients are out-patients of the hospital; and we cannot help thinking that Sir Sydney would better have forwarded the objects of the Commission and the interests of the public by stating frankly that Bartholomew's treated in 1889, 156,400 out-patients, rather than 19,001 out-patients and 137,399 casualties.

Unless St. Bartholomew's is different in this respect from other hospitals, the relation between the out-patients and the senior students with regard to treatment was not put before the Commission quite so clearly or so frankly as it should have been by Sir Sydney Waterlow. He was confronted by Mr. Nelson Hardy's statement that "when the pressure of business becomes great, a number of cases, sometimes cases that have been seen before, but sometimes also cases that have not been seen before, are seen by senior students, and are seen only by senior students." "That can never occur," said the witness, "except in direct contradiction to the instructions of the governors; and, speaking from my own personal experience, it is an erroneous statement of what takes place." This is a point to which the Commissioners would do well to address themselves with some resolution. It is important to ascertain whether or not the custom of permitting medical students to examine and prescribe for out-patients prevails to any considerable extent; and the only way of really settling the point is to obtain the testimony of a large number of witnesses.

Sir Sydney Waterlow expressed decided disapprobation of the practice which prevails in some places of mixing up paying with non-paying patients. This is a question which has been much debated both in this country and elsewhere, and the discussion has been of great service. Sir Sydney says nothing against pay hospitals as such; nor indeed is there anything to be said, so long as the patients pay such a sum as will adequately remunerate the doctors as well as defray all other necessary expenses. Free hospitals, the witness thinks, should be free altogether; and after much anxious consideration of the question in all its bearings, we are inclined to agree with him. Many people affirm that the purely gratuitous treatment of hospital patients has the effect of pushing them some way down the hill towards pauperism. Perhaps it has. But what else can be done with a sick person who has little or no money, except to try to make him well again so that he may earn money, it is not easy to see. No doubt the provident system theoretically, at any rate, should be his medical providence. But up to the present time, except in very rare instances, the provident system has been a comparative failure. The reason of this probably is that the pay offered to medical officers of provident institutions is not such as to tempt really able men to devote time and skill to them. If that be so, the provident system must be altered, or the poor will continue to go to hospitals, and the rich will encourage them to do so, whether pauperisation be the result or not.

On the important subject of medical qualifications, Sir Sydney Waterlow was rather put into a corner. He was obliged to admit that practically the graduates of two colleges, the College of Physicians of London and the College of Surgeons of London, have a monopoly of the higher medical and surgical appointments at St. Bartholomew's Hospital. He stated, however, that there was nothing in the original charter of the institution rendering this arrangement imperative. On the contrary, it seems to have reached its present stage as the result of exceedingly natural development. The practitioners practising in London and holding appointments at the hospital have done their best from time to time to keep competitors from other medical schools at a distance by disqualifying them beforehand. Sir Sydney acknowledged that the regulations of the hospital had been set at defiance in the case of Dr. Mathews Duncan, the famous obstetrician, who, when appointed to his present post, had no London qualification whatever. The witness seemed to think that it was not very creditable to the statesmanship of the Bartholomew's managers that they should shut out from their honorary appointments the great English, Scotch, and Irish Universities, unless the holders of the degrees of those universities were rendered competent for appointments at Bartholomew's by the two London colleges. When hard pressed by the Commissioners, Sir Sydney said that personally he had an open mind on the subject; and he did not doubt that if public opinion demanded it the managers would resolve upon improved legislation in this regard.

The evidence of Sir Sydney as to the influence of the Hospital Sunday Fund in promoting uniformity of accounts and otherwise helping to discourage extravagance and to increase efficiency, was full and valuable. There is no doubt that a uniform method of keeping accounts at hospitals would greatly facilitate the work not only of the professional auditor, but also of the newspaper critic and of the benevolent public. The Hospitals Association has done much to bring about uniformity, and though Sir Sydney Waterlow spoke with cautious hesitancy, there is reason to believe that both he and the Sunday Fund managers would be glad if this much-needed reform could be effected. In our judgment hospital managers would be well advised to take the initiative in such a desirable and advantageous movement.

